# Correlates of Stress in the College Environment Uncovered by the Application of Penalized Generalized Estimating Equations to Mobile Sensing Data

**DOI:** 10.2196/12084

**Published:** 2019-03-19

**Authors:** Alex W DaSilva, Jeremy F Huckins, Rui Wang, Weichen Wang, Dylan D Wagner, Andrew T Campbell

**Affiliations:** 1 Department of Psychological and Brain Sciences Dartmouth College Hanover, NH United States; 2 Department of Computer Science Dartmouth College Hanover, NH United States; 3 Department of Psychology Ohio State University Columbus, OH United States

**Keywords:** psychology, stress, mobile sensing, college campuses

## Abstract

**Background:**

Stress levels among college students have been on the rise for the last few decades. Currently, rates of reported stress among college students are at an all-time high. Traditionally, the dominant way to assess stress levels has been through pen-and-paper surveys.

**Objective:**

The aim of this study is to use passive sensing data collected via mobile phones to obtain a rich and potentially less-biased source of data that can be used to help better understand stressors in the college experience.

**Methods:**

We used a mobile sensing app, StudentLife, in tandem with a pictorial mobile phone–based measure of stress, the Mobile Photographic Stress Meter, to investigate the situations and contexts that are more likely to precipitate stress.

**Results:**

Using recently developed methods for handling high-dimensional longitudinal data, penalized generalized estimating equations, we identified a set of mobile sensing features (absolute values of beta >0.001 and robust *z*>1.96) across the domains of social activity, movement, location, and ambient noise that were predictive of student stress levels.

**Conclusions:**

By combining recent statistical methods and mobile phone sensing, we have been able to study stressors in the college experience in a way that is more objective, detailed, and less intrusive than past research. Future work can leverage information gained from passive sensing and use that to develop real-time, targeted interventions for students experiencing a stressful time.

## Introduction

Stress levels among college students have been on the rise for years [1]. According to a recent large-scale nationwide survey, rates of student stress continue to climb as more than half of students (57%) reported experiencing “more than average stress” [2]. In the workplace, it has been conservatively estimated that stress costs the United States economy up to US$125 billion annually [3]. Although not directly measured in dollars and cents, evidence for the negative impact of stress on college students can be observed through hampered academic performance, as 30% of students noted that stress caused them to receive a lower grade on an exam or course, fail or drop a course, or it interfered with thesis or practicum work [2].

Broadly, stress can be defined as any sort of negative event related to demand, threat, or harm. Following a stressful event is a biological and behavioral response geared toward altering the present situation or otherwise adapting to the stressor [4]. Along with hindering academic performance, stress is thought to be related to a host of negative emotional states and health outcomes, including depression, obesity, and cardiovascular disease [5-7]. Indeed, as levels of stress have risen among college students, so have issues related to mental health [2]. Not surprisingly, a survey of college counseling center directors indicated that a clear majority (90%) of centers reported an increase in severe psychological problems among students [8].

A primary source of stress for students is academics. Stress because of class attendance and homework were among leading stressors in one study of college students [9]. Further, research suggests that in addition to academic performance, students are also stressed by a pressure to succeed and about concerns regarding postgraduate planning [10]. Of course, in the college environment, stressors other than those academic in nature also exist. Interpersonal aspects of a student’s life, such as worrying about forming friendships, fitting in socially, and getting along with roommates, were reported as stressful elements by students [11]. In addition, environmental sources of stress, such as financial difficulties, and changes in eating and sleeping patterns also contribute to levels of student stress [11,12].

Until recently, the dominant method for sampling information about students’ lives came by way of daily and weekly diaries where details about students’ thoughts, experiences, behaviors, and activities could be recorded over a specified time range. Indeed, approaches such as this have shed light on various aspects of a college students’ life, including interpersonal student relationships, drinking behavior, and coping with stressors [13,14]. However, diary methods can be time intensive for participants and typically rely entirely on retrospective self-report of activities, a task that is known to be susceptible to various forms of bias—chiefly a bias due to cognitive and memory limitations [15]—resulting in inaccurate reports of the frequency and severity of events.

With the widespread adoption of mobile phones in recent years, new approaches have been developed to use mobile phones for studying real-time or nearly real-time behaviors and attitudes [16]. Mobile phones can be used to collect both ecological momentary assessments (EMAs), such as a brief stress inventory, and automatic passive sensing data (eg, audio and location data collected via microphones and phone GPS). Researchers have successfully related mobile phone sensing features to mental health in a clinical setting [17,18]. Wang and colleagues [19] were among the first to adapt this technology to better understand student mental health with the creation of the StudentLife continuous sensing app. With the StudentLife app, researchers have been able to capture daily fluctuations in students’ mental and emotional well-being by linking passive sensing features (eg, sleep, location, social interaction) to aspects of standard health-related questionnaires [19-21].

Based on prior research using primarily self-report measures, aspects of the college experience are clearly stressful for students ranging from interpersonal relationships to academic achievements. However, one issue with this work is that these measures are often obtained once per term and are thus subject to the limitations and biases of retrospective self-report measures, which makes it difficult to paint an accurate picture of how stress manifests itself in the daily lives of students. Recently, a proof-of-concept study using passive mobile phone sensing investigated the relationship between passive sensing measures and various mental health outcomes (eg, depression, loneliness, stress) [20]. This proof-of-concept study demonstrated the utility of mobile phone sensing apps despite having focused on a relatively small number of sensing-derived features. Here, we expand on this work by focusing on a considerably larger number of passive sensing features to comprehensively categorize a day in the life of a college student and also make use of recently developed advances in modeling, such complex, longitudinal data. As examples of the mobile sensing features become available, inferences can be made about the quality and quantity of an individual’s interpersonal relationships by conversation features or phone usage, about academic-related activity by time spent in study locations, about sleep through a combination of sensing features (eg, periods of movement, presence of light, phone usage), about exercise by the time spent in gymnasiums, and so on. Moreover, these features can be examined not only daily, but also at a finer grain within a day. Looking at the data more minutely could help fingerprint meaningful behavioral patterns in relation to stress. For example, it could be the case that phone usage decreases dramatically during evening hours when stressed, but not globally across the day. Thus, the purpose of this study is to further the understanding of stress dynamics on college campuses by leveraging a dataset rich in passive sensing features to accurately, and naturally, capture possible stressors in the lives of students. Whereas prior work has largely focused on a small number of features, we are able to use an array of mobile sensing features and then subsequently analyze these data with recently developed methods that both account for statistical dependencies inherent in longitudinal data and perform feature estimation and selection on high-dimensional data [22]. By doing so, we are able to identify a collection of mobile sensing features across the domains of social activity, location, and ambient noise that are related to stress levels across the academic term.

## Methods

### Participants

Data were collected from 95 participants who agreed to provide mobile sensing data across the winter or spring terms. Demographic information was missing for one participant. Of the 94 participants with complete demographic information, 56% percent were female (53/94). The mean age of participants was 21 (range 18-28) years. This study was approved by the Dartmouth Committee for the Protection of Human Subjects.

### Mobile Sensing

The StudentLife app was used to collect sensing data and to administer EMAs; a version of the app exists for both Android and iOS operating systems. The app continuously collects and records students’ sleep, physical activity, phone usage, location, and sociability data in addition to randomly administering EMAs probing stress once a day. Data from StudentLife is uploaded to a secure server (encrypted and transmitted through HTTPS) whenever a participant is both using WiFi and charging their phone, which they were encouraged to do daily.

#### Conversations and Ambient Sound

Social interaction was measured by the number of independent conversations and their respective durations. Critically, to protect participants’ privacy, raw conversation data was never recorded or analyzed. Instead, relevant features were extracted from the audio stream and used to identify the presence of a human voice and infer a human voice and conversation patterns. In this way, the number of conversations along with their respective durations were calculated. These conversation-related features, along with features related to ambient sound, were subsequently uploaded to a secure server [19,23].

#### Sleep

Sleep features were inferred through a combination of passive sensing features (ambient light, audio amplitude, movement activity, screen on/off). In this way, three features were computed: sleep onset, wake time, and sleep duration. This measure of sleep has been shown to be accurate within +/–30 minutes for total sleep duration [19].

#### Location

Density-based spatial clustering of applications with noise (DBSCAN) [24] was used to cluster GPS coordinates to uncover where students were spending a significant amount of time. Every building on campus was mapped with respect to its primary function; thus, the amount of time a participant spent at locations such as dining centers, the gym, and study locations could be measured along with their total distance traveled and the number of different places visited. The institution where this study was conducted was an ideal environment for extracting location-based data because more than 90% of students live on campus and first first-year students are required to reside on campus during their first year of school. Further, all students are required to have a campus meal plan for the entirety of their education.

#### Mobile Phone Usage

The total number of phone lock and unlock instances was computed along with the total duration a phone was unlocked during the day.

#### Epochs

There is a large amount of daily variability in a student’s schedule; thus, we also looked at data not only over the course of a day but within the following three epochs: 9 am to 6 pm (day), 6 pm to 12 am (evening), and 12 am to 9 am (night). Accordingly, we could estimate and add the relative occurrences of behaviors within each epoch compared to their daily totals as features.

In total, across the different sensing-based features (conversation, sleep, location, and phone usage) and within the previously mentioned time epochs (9 am-6 pm, 6 pm-12 am, 12 am-9 am), 60 passive sensing variables were computed.

### Measures

**Figure 1:**
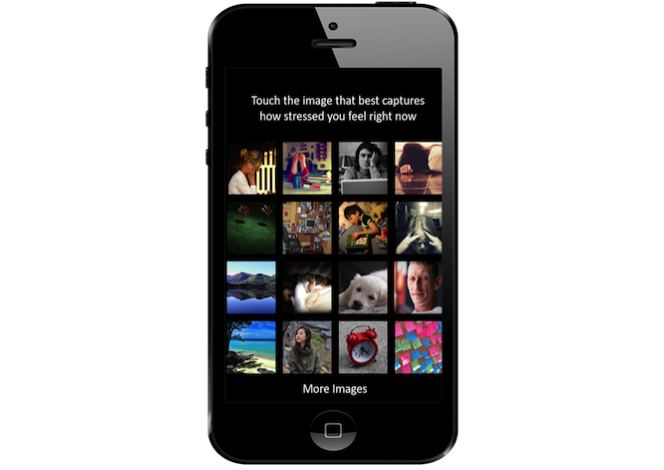
The Mobile Photographic Stress Meter (MPSM) is a pictorial, user-friendly way to measure stress.

The Mobile Photographic Stress Meter (MPSM) was used to measure stress (Figure 1) [25]. The MPSM is a series of 16 images depicting varying levels of stress (1 depicts a relaxing beach, and 16 displays someone on the verge of breaking down). The user simply taps the image that best describes their current stress level. Critically, usability is an important aspect of any mobile phone app. In a pilot study, along with demonstrating item validity, the majority of MPSM users enjoyed using the mobile stress meter, reporting comfort, ease of use, and an overall positive impression of the app [25]. In addition, participants were asked to indicate their level of stress on a scale from one (not at all) to five (very much); this stress scale was not used in the main analyses but was used to validate the relationship between stress and the MPSM.

### Analyses

Datasets across a variety of disciplines are quickly increasing in complexity and dimensionality with the onset of new data collection technologies. In typical studies, the number of observations is much larger than the number of features or covariates. When referring to high-dimensional data in this context, the number of features nears or even reaches the number of observations. In addition, these datasets become even more complex when collected over time. Recently, researchers have proposed a technique to estimate models for longitudinal data with high-dimensional covariates [26]. More specifically, Wang and colleagues [26] combined techniques used for analyzing clustered and longitudinal data (ie, generalized estimating equations [GEE]), an extension of the generalized linear model to accommodate clustered and longitudinal data, with penalized regression methods. This resulted in a technique known as penalized generalized estimating equations (PGEE). An advantage of these recently developed techniques is that, in contrast to previous work that focused on a few features out of many possible mobile sensing features, this technique enables us to use a greater number of mobile sensing features and feature elimination to prune uninformative ones. In this study, PGEE were used to simultaneously estimate and select features from the 61 (all the sensing features plus a time variable) variables contributing to stress with the package PGEE in the R environment [22,26,27]. Like GEE, PGEE fit a marginal regression model to the data and require the selection of a working correlation matrix; further, it can yield consistent estimates even if the working correlation structure is incorrectly specified [22,26]. Here, the independence correlation matrix was used as the working correlation matrix, and the smoothly clipped absolute deviation (SCAD) penalty was used as the penalization function due to its efficiency and lack of bias compared to other penalization techniques (eg, least absolute shrinkage and selection operator [LASSO]) [22,28]. To select the optimal tuning parameter, five-fold cross-validation was implemented. Additionally, robust variance was calculated for effective inference.

## Results

**Figure 2:**
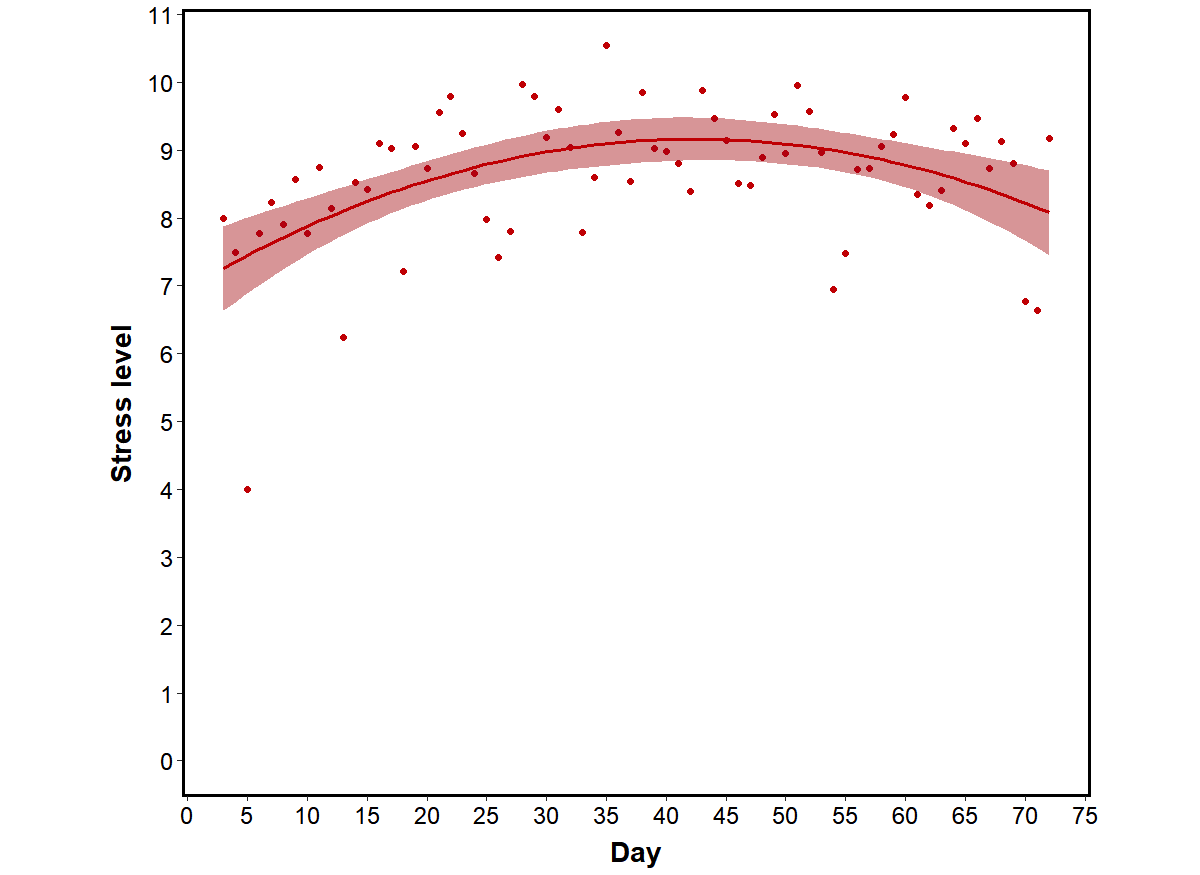
Average daily stress over the course of the term. The shading represents the 95% confidence interval around the fitted values.

Before any analyses, the data were cleaned to remove participants who encountered technological complications with their mobile phone or who failed to respond to more than 50% of the MPSM survey used to measure stress. Additional days were discarded when extreme outliers were observed in the location data (distance >0.975 quantile). The aim here was to remove days when students may have taken a day trip during the term; spending the majority of the day traveling severely skewed location-based data. After this step, the remaining 72 participants had 43.1 days of data on average (SD 11.8). Across these participants, the overall rate of failure to respond to the MPSM survey prompts was 18.57% (576/3101). Next, with the cleaned dataset the relationship between stress (self-reported, range 1-5) and the MPSM was assessed using a mixed-effect model with random subject intercepts. Supporting the MPSM pilot data, MPSM and self-reported stress were strongly related (beta=2.037, SE 0.076, *P*<.001) providing further validity for the MPSM as a measure of stress. Of general interest was understanding how reported stress unfolded across the term. Thus, the relationship between stress and time was also assessed. A mixed-effect analysis incorporating fixed linear and quadratic time effects, random subject intercepts, and random slopes for both the linear and quadratic effects of time revealed a significant linear effect (beta=1.282, SE 0.509, *P*=.02) and a significant quadratic effect (beta=–1.203, SE 0.491, *P*=.02) of time on the MPSM measure of stress, indicating that stress increased across the term but also experienced a bump near the middle of term presumably reflecting stress brought on by midterm exams (Figure 2). This quadratic effect is similar to the patterns observed in past research [20].

**Figure 3:**
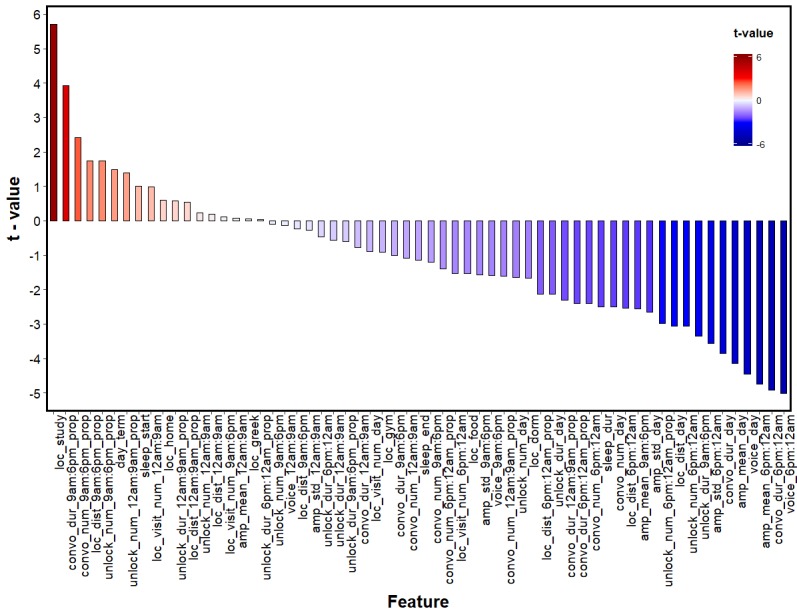
Relationship between stress and sensing features using t values to depict the relationship in a pairwise fashion. Amp: amplitude; convo: conversation; day: sensing data across an entire day; dist: distance; dur: duration; loc: location; num: number; prop: proportion of sensing data occurring within a time period; std: standard deviation.

Correlation matrices are used to visually assess relationship patterns between variables in high-dimensional data. However, in this dataset, looking at correlations between sensing features may be misleading due to the clustered and unbalanced nature of the data. Thus, Figure 3 was created to aid in visualizing the relationship between stress and the set of sensing features. The estimates in the figure represent the *t* values from a series of pairwise mixed-effect analyses regressing stress with each of the variables in the dataset. From Figure 3, one can see a few themes emerging. For example, it appears that the majority of sensing features seem to be inversely related to stress. Generally, it also appears that a variety of sensing features see a decrease in usage/occurrence during the evening epoch when stress is high.

Unlike mixed-effect models, GEE (and, as a result, PGEE) are not “full-likelihood” models [29]. Thus missing data must be accommodated differently, here imputed before model fitting. Missing MPSM data points were imputed using Kalman smoothing. Each subject’s trajectory of MPSM scores was treated as a structural time series model and Kalman smoothing was then used to impute the missing values in the time series [30]. In the resulting completed dataset, 10 skewed covariates were transformed using log or log(x+1) transformations (eg, distance traveled, unlock duration). Finally, the outcome variable and covariates were standardized [31] and analyzed using PGEE. Of the 61 features used in the analysis, six were found to be significantly related to levels of stress by the PGEE analysis. Time spent in dining centers (beta=–0.024, robust *z*=–2.968), distance traveled between 6 pm and 12 am (beta=–0.021, robust *z*=–2.670), and mean audio amplitude between 6 pm and 12 am (beta=–0.031, robust *z*=–2.074) were found to be inversely related to stress, whereas time spent in study locations (beta=0.071, robust *z*=3.569), proportion of time spent conversing between 9 am and 6 pm (beta=0.030, robust *z*=2.706), and the proportion of conversations occurring between 9 am and 6 pm (beta=0.021, robust *z*=2.616) were found to be positively associated with stress (Table 1).

**Table 1:** Features related to stress as selected by the penalized generalized estimating equations (PGEE) analysis.

Feature	Estimate	Robust standard error	Robust *z*
Time in study locations	0.071	0.020	3.569
Proportion of conversation duration from 9 am-6 pm	0.030	0.011	2.706
Proportion of conversation number from 9 am-6 pm	0.021	0.008	2.616
Distance traveled from 6 pm-12 am	–0.021	0.008	–2.670
Time in food locations	–0.024	0.008	–2.968
Mean audio amplitude 6 pm-12 am	–0.031	0.015	–2.074

## Discussion

### Principal Findings

Using the MPSM and continuous passive sensing, we were able to measure stress and richly sample the daily life of a college student. From the resulting dozens of features, we were able to select the features best predictive of stress while accounting for the clustered nature of the data. The finding that time spent in study locations was the strongest predictor of stress provides a novel and unobtrusive index to assess school-related strain and meshes well with past research indicating that academics were a serious stressor for students [9,10]. We were also able to identify an environmental factor identified by past research related to stress [11,12] as students were spending less time in food locations when reporting greater amounts of stress. Further, we were able to uncover novel aspects of movement and speech patterns that were associated with stress. In the evening, stressed students were around less noise and showed reduced patterns of movement between 6 pm and 12 am. During the day, stressed students spent a greater proportion of time conversing between 9 am and 6 pm, and they exhibited a greater proportion of conversation between 9 am and 6 pm.

Of note was that four of the six variables selected related to specific time epochs, which underscores the importance of looking at these data not only aggregated across a day but at finer scales within a day. For example, past work did not observe a significant relationship between daily stress levels and speech duration across the day, although there was a modest relationship between the two measures [20]. It may have been the case that a relationship similar to the association we observed between stress and relative conversation between 9 am and 6 pm was present but masked by aggregating the data across the day.

From these data, we can more accurately begin to conceptualize what a “stressful” day looks like for a college student. Over the course of the day, students are eating less and spending more time in study locations. They are around more conversation during the day while they move to and from class, but are not socializing with others during the evening, a time that many would consider more leisurely. In lieu of socializing during evening hours, students are spending time in a quiet place, possibly engaged in studying or other sedentary pursuits. Put differently, it could be the case that being around others during the evening shields students from stress, a finding that fits well with the notion that social support moderates life stress levels [32].

Finally, by using PGEE, we were able to take advantage of a complex dataset. This technique has many applications beyond examining passive sensing data and may be of use to any researcher who has a large dataset collected over time. As the authors of the R package PGEE mention, large longitudinal datasets are becoming commonplace in fields such as health, economics, genomics, and the behavioral sciences. To illustrate PGEE, the authors apply their method to a yeast cell cycle gene expression dataset to identify factors that play an important role in the transcription of genetic information from DNA to mRNA [22,26]. Further use cases for PGEE could be realized in longitudinal genomic studies where genetic data are collected and single nucleotide polymorphisms are related to some sort of phenotype outcome at the person level (eg, breast cancer or asthma). At a level higher than the genome, PGEE could also be of use to those in the realm of health economics. For example, determining the most important predictors of life expectancy in a country over time from a large number of demographic, socioeconomic, and cultural variables. Finally, PGEE could be applied to future passive sensing projects in the mobile health domain. For instance, mobile sensing could be paired with wearable technology (eg, a smart band) to uncover what physiological and environmental variables precipitate cigarette craving.

### Limitations and Future Directions

Although the purpose of this study was to examine stress in a particular population (college students), future studies may want to include individuals from a variety of institutions and age groups (eg, high school or workplace), which could provide valuable insights into boosting education quality and workplace productivity. A strength of this study was that it was conducted at a relatively small, self-contained college campus that has an extremely high number of students living on campus; however, it could be challenging to take this project to scale at a larger, more urban university where the number of students living off campus is much higher. An additional limitation of this study is that the granularity of location data does not permit strong inferences concerning student activities while in locations such as libraries. Although we know students in a library are most likely studying, college libraries are large buildings; they could be socializing or working more casually. Another interesting avenue to explore would be the placement of Bluetooth beacons within buildings to expand the spatial resolution of the location data. Bluetooth beacons would allow one to detect whether a student is working in an open, causal section, a quiet-only zone, or to discover whether a student may have rented out a private cubicle.

### Conclusion

In sum, we used a picture-based measure of stress (MPSM) alongside modern mobile phone sensing technology to gain a better understanding of stressors affecting college students. By taking this approach, we have been able to study stressors in the college experience in a way that is more objective, detailed, and less obtrusive than past research. With this knowledge, future work can leverage information gained from passive mobile sensing and use that to develop real-time, targeted interventions for students experiencing increased stress.

## Abbreviations

EMA: ecological momentary assessment
GEE: generalized estimating equations
MPSM: Mobile Photographic Stress Meter
PGEE: penalized generalized estimating equations
